# A Method for in Situ Interfacial pH Detection

**DOI:** 10.1021/acs.jpclett.5c02002

**Published:** 2025-08-21

**Authors:** Karina N. Catalan, Aaron D. Ratschow, Hans-Jürgen Butt, Kaloian Koynov

**Affiliations:** Max Planck Institute for Polymer Research, 55128 Mainz, Germany

## Abstract

Functionalized silica-based surfaces are widely used across industries, from semiconductors to pharmaceuticals. Aminosilanes are commonly employed as coupling agents during surface functionalization to anchor diverse functional molecules. However, the surface modifications perturb interfacial physicochemical properties, resulting in a significant shift in interfacial pH compared to the bulk solution. This shift complicates direct measurement and accurate monitoring of interfacial conditions. To overcome this challenge, we functionalized glass surfaces with aminosilane-coupled pH-sensitive fluorescent dyes and utilized confocal microscopy to measure their fluorescence response to changes in bulk pH. Complementing these experiments, we developed a theoretical model describing equilibrium surface chemistry taking into account electrostatic interactions at aminosilane-functionalized glass interfaces. It revealed a linear relationship between interfacial and bulk pH, with the interfacial pH varying over a narrowed range compared to the bulk pH. Building upon these insights, we calibrate the fluorescence response of the grafted pH-sensitive dyes. This integrated approach enables precise and reliable in situ monitoring of interfacial pH under various conditions, demonstrating significant potential for environmental sensing and advanced material characterization.

## Graphical abstract

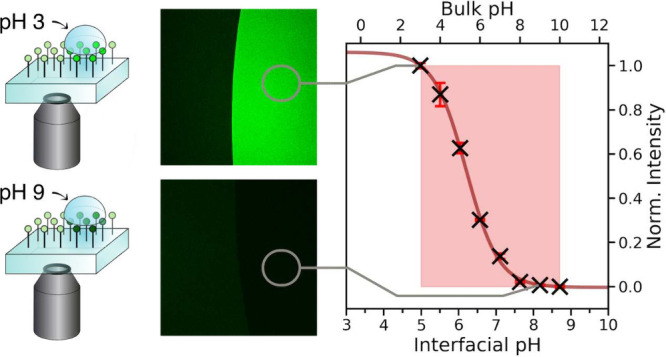

Surface functionalization to impart specific chemical, physical, and biological properties has become a crucial area of research in developing advanced nanomaterials,^1–4^ smart surfaces,^5,6^ biocompatible interfaces,^7,8^ and electronics.^9–11^ A common application involves environmental sensing,^12–16^ where fluorescent dyes are grafted onto nanoparticles or surfaces to label specific components and track changes in environmental properties.^17,18^ Silica-based materials are widely used for these applications because they are easy to functionalize, they are chemical stable, biocompatible, and have beneficial optical properties.^19^ Silica-based materials, such as glass or silicon oxide, expose hydroxyl groups^20^ suitable for attaching pH-sensitive dyes via coupling agents. Aminosilanes are among the most widely studied.^21–24^

When a pH-sensitive dye is grafted onto a surface and brought into contact with an aqueous electrolyte solution, chemically active surface groups undergo protonation or deprotonation.^25^ The equilibrium protonation state of the dye, which governs its optical response, is described by the Henderson–Hasselbalch equation^26^ and determined by the local, interfacial pH (pH_int_) and the dissociation constant (p*K*) of each species. The p*K* value, in particular, can vary due to steric effects arising from grafting or bonding.^27,28^ The surface charge resulting from dissociated surface groups leads to the formation of an electric double layer,^29,30^ which in turn influences the local proton distribution and causes a shift between the bulk and interfacial pH.^31^

Translating changes in the dye’s fluorescent response to quantitative values for interfacial pH requires a profound understanding of this pH shift. Several studies have attempted to quantify the shift by using fluorescence titration experiments^32^ and computational simulations^31,33^ to explore chemical and electrostatic interactions at the interface. These efforts are often complemented by surface characterization techniques, including ζ-potential measurements^32,34^ and X-ray photoelectron spectroscopy (XPS).^35^ The ζ-potential is the electric potential at the shear plane. It is determined by the chemical distribution and experimentally accessible through electroosmotic flow techniques. Using XPS, the chemical distribution at the solid surface can be measured.

Based on the site binding model proposed by Yates et al.,^36,37^ Behrens and Grier^38^ successfully modeled the interplay between the chemical equilibrium and the electrostatic double layer for simple silica surfaces. Similar models have found use in gated electroosmotic flow^39,40^ and gated nanoparticle traps.^41^ These approaches combine the chemical equilibrium, described above, with the Gouy–Chapman-Stern model to represent the structure of the electric double layer and the subsequent pH shift.

However, for more complex functionalized surfaces, there is still a lack of a unified model that integrates all relevant phenomena in equilibrium to accurately explain and predict the structure of the electric double layer.

The aim of this work is to quantitatively measure interfacial pH on silica-based surfaces. To obtain the local pH, we functionalized the surfaces with a pH-sensitive fluorescent dye and measured the fluorescence intensity by confocal microscopy. The recorded intensity was then converted to interfacial pH values through a calibrated fluorescence–pH response curve. We developed a theoretical model that accounts for all dissociable surface groups and the electrical double layer to predict the shift between bulk and interfacial pH. The model reveals a linear relationship between the bulk and interfacial pH. Building on this, we developed a simple method for calibrating the fluorescence response of grafted pH-sensitive dyes that enables real-time, quantitative, in situ monitoring of interfacial pH. Our approach addresses critical gaps in existing methods and provides a robust tool for understanding interfacial chemistry, with applications ranging from biosensor calibration to designing smart coatings for industrial applications.

To graft pH-sensitive fluorescent dyes onto glass surfaces we first functionalized borosilicate glass microscope coverslips with 3-aminopropyltriethoxysilane (APTES) or 3-aminopropyldimethylethoxysilane (APDMES) as coupling agents. After hydrolysis of the ethoxy groups, which are converted into silanol groups (−Si–OH), they react with hydroxyl groups (−OH) present on the glass surface, forming stable, covalent Si–O–Si bonds.^42^ Then, we grafted a pH-sensitive dye (pHrodo iFL Green STP Ester, ThermoFisher) onto the functionalized glass surface. In the grafting reaction a covalent amide bond is formed between an exposed amino (−NH_2_) group on the surface and a 4-sulfo-2,3,5,6-tetrafluorophenyl (STP) ester group of the dye.

The density and arrangement of aminosilane coverage are influenced by the specific type of aminosilane, determining the number of ethoxy groups available for condensation, and the silanization protocol.^43^ We use APDMES and APTES in a toluene solution (TS),^43^ as well as APTES through chemical vapor deposition (CVD).^43,44^ This resulted in the three different types of functionalized glass surfaces (Figure 1), which vary in the ratio of surface chemical species, hydroxyl:amino:dye (SI S1).

**Figure 1:**
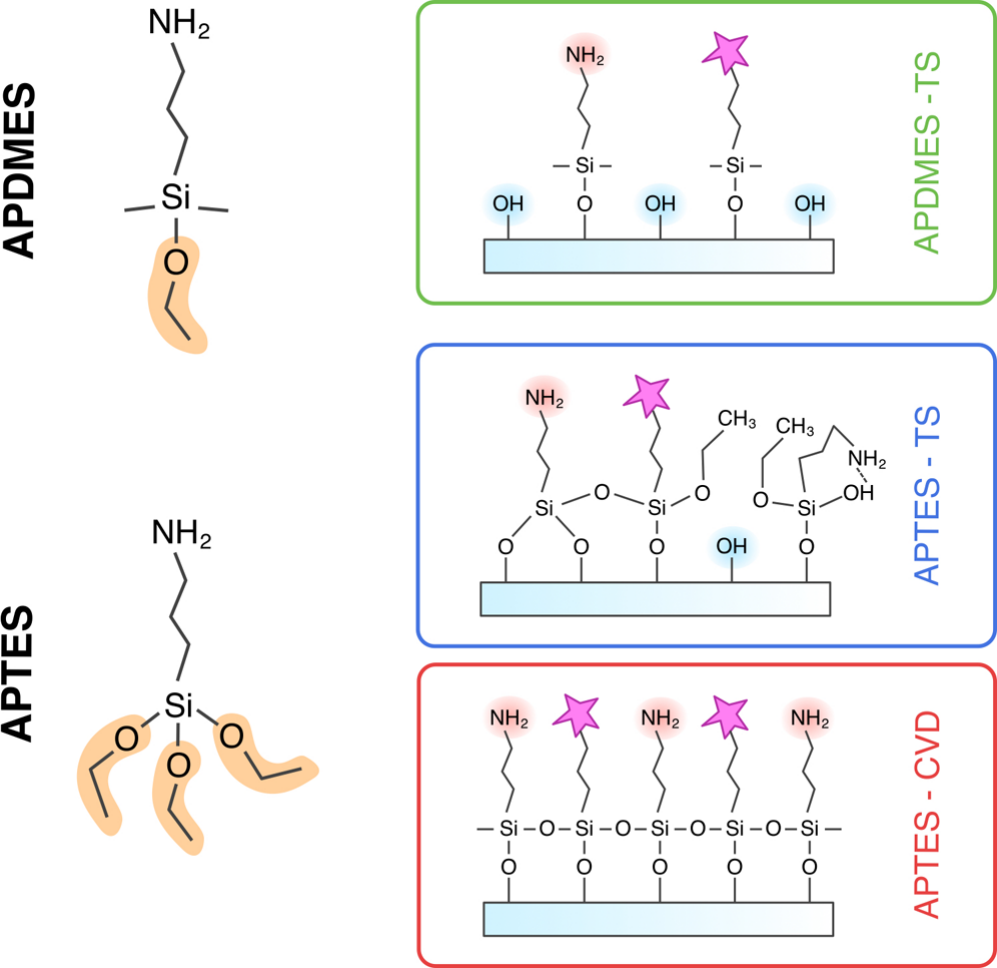
Chemical structure of APTES and APDMES and their structure when grafted to the surface via different silanization protocols such as toluene solution (TS) and chemical vapor peposition (CVD). Subsequently the dye (pink star) is grafted on top and substitutes of some of the amino groups.

Coating glass with APDMES in a toluene solution (APDMES-TS) leads to a relatively low degree of hydroxyl group substitution^43^ due to its single ethoxy group, which limits its reactivity with surface hydroxyls. In contrast, APTES, with three ethoxy groups, enables a higher degree of substitution. However, APTES in a toluene solution (APTES-TS) can yield less uniform surface structures due to aggregation and variability in condensation.^45^ Using chemical vapor deposition (APTES-CVD) results in a more homogeneous layer, as this method minimizes solvent effects and provides a controlled environment for uniform surface coverage.^43,46^

To characterize these surfaces, the surface topography and roughness were first evaluated by AFM (SI S2) to assess the smoothness of the functionalized layers. In parallel, we assessed the impact of different functionalizations on surface charge density by measuring the ζ-potential versus pH (titration). Surface ζ-potential measurements were performed by means of streaming potential and streaming current methods^47^ for all samples (SI S3), both before and after dye grafting, as shown in Figure 2, and the isoelectric points (pI) were determined (Table 1).

**Table 1:** Isoelectric Points (pI) of All Samples

Aminosilane^a^	pI_1_ ^b^	pI_2_ ^b^
APDMES-TS	4.4	4.0
APTES-TS	5.7	5.3
APTES-CVD	7.0	6.4

a This accounts for the aminosilane and silanization protocol used for each sample..

b Isoelectric points (pI) before (pI_1_) and after (pI_2_) dye grafting.

**Figure 2:**
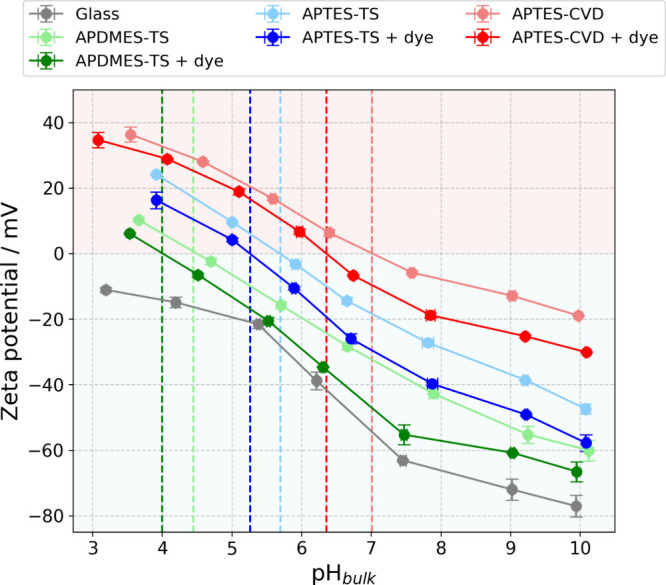
ζ-potential titration curves for functionalized samples with aminosilanes, with and without subsequent dye grafting, including cleaned glass as a reference. The isoelectric points (pI) are indicated. The conductivity remains nearly constant in the pH range 4–10, at 12.5 ± 2.5 mS/m.

Titrations were initiated at pH 10 using a KCl solution with a conductivity of 12.5 mS/m and progressively titrated with HCl to reach pH values around 3–4 (see Figure 2). Conductivity was recorded at each pH point during the titrations. Theoretical calculations of the conductivity, based on the initial conditions of the KCl solution and the titrant, were performed to compare to the measured conductivities, allowing a theoretical estimation of the Debye length κ^–1^ at each point, ranging from 9.75 to 11.3 nm (SI S4).^48^

The fluorescence response of surface-tethered dyes differs from that in free solution. To convert the fluorescence intensity into interfacial pH, we account for the coupling of proton dissociation and electrostatic interactions at the interface.^49–51^ To this end, we consider the behavior of the functionalized surfaces. All chemical species present on the glass surface — hydroxyl, amino, and dye groups — can undergo dissociation in contact with an aqueous electrolyte solution.^52^ They can accept or donate a proton depending on the interfacial pH and their specific dissociation constant (p*K*). Upon association/dissociation, the species tethered to a reactive site on the surface (S) acquire a charge: hydroxyl and dye groups become negatively charged when deprotonated,^32^ while amino groups acquire a positive charge when being protonated,^53^

$$\begin{array}{c} S\text{−}\mathrm{OH}⇌S\text{−}O^{-}+H^{+}\left(\mathrm{hydroxyl}\right)\\ S\text{−}{\mathrm{NH}}_{3}^{+}⇌S\text{−}{\mathrm{NH}}_{2}+H^{+}\left(\mathrm{amino}\right)\\ S\text{−}\mathrm{DH}⇌S\text{−}D^{-}+H^{+}\left(\mathrm{dye}\right) \end{array}$$

The ratio of deprotonated to protonated forms of each species at a given pH is governed by the Henderson-Hasselbalch equation.^54^ While in solutions, the bulk pH determines the dissociation equilibrium. For functionalized surfaces it is the interfacial pH (pH_int_).

Our model predicts the potential drop across the electric double layer and derives other relevant parameters from the combined Gouy-Chapman-Stern model^55^ and the law of mass action.^56^ The model can be applied to all samples containing the same functional groups across a wide pH range, and considering the ionic strength of the electrolyte solution.

Our approach considers three types of chemical species at the surface: hydroxyl, amino, and dye groups. Each can exist in its associated/dissociated form. The species are assumed to be homogeneously distributed and strictly localized on the glass surface, as illustrated in the schematic in Figure 3.

**Figure 3:**
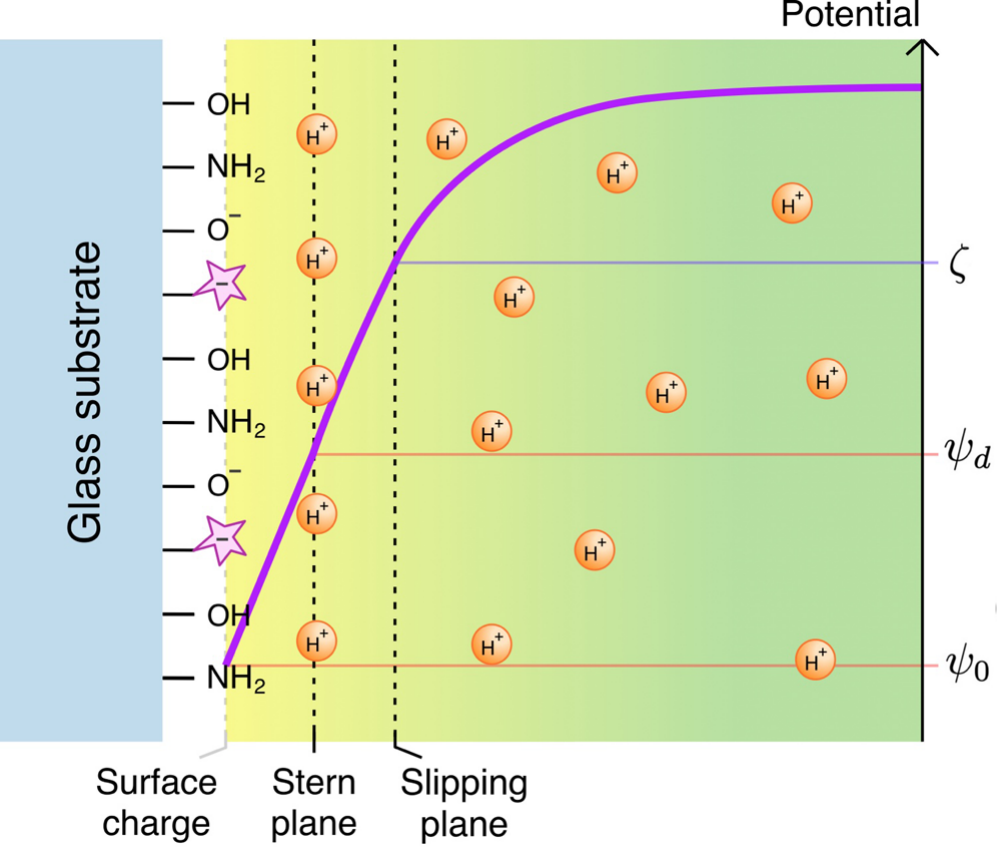
Schematic representation of the electric double layer (EDL) on a pH-sensitive dye-functionalized glass substrate in contact with an electrolyte solution. The diagram illustrates the distribution of surface charge, the Stern plane, and the slipping plane, along with the potential profile (ψ_0_, ψ_*d*_, and ζ-potential) across the interface.

Each functional group at the interface distributes between its acidic and basic forms following

$$\frac{{\left[H^{+}\right]}_{\mathrm{int}}\Gamma_{i^{-}}}{\Gamma_{Hi}}=10^{-{\mathrm{pK}}_{i}}M$$ 1

where *i* runs over all dissociable species, [H^+^]_int_ = 10^–pH_int_^M is the proton activity at the interface, Γ_*i* ^–^_ is the density of basic (deprotonated) groups, Γ_H*i*_ is the density of acidic (protonated) groups, and p*K*_*i*_ is the dissociation constant (values taken from literature, SI S5).^38^ Depending on the species, some of these states will carry a charge. By considering the sum of all charged species at a given pH value, weighted by their valencies, we determine the net surface charge density σ,

$$\sigma =-e\Gamma_{O^{-}}+e\Gamma_{{{\mathrm{NH}}_{3}}^{+}}-e\Gamma_{D^{-}}$$ 2

This net charge at the surface leads to the spontaneous formation of an electric double layer.^57^ The electric double layer consists of the bound surface charge density σ, an adsorbed monolayer of partially hydrated countercharges in the Stern layer, and a cloud of diffuse countercharges in the diffuse layer. The surface potential ψ_0_, associated with the surface charge density σ, drops across the electric double layer. Across the Stern layer, between the solid surface and the Stern plane, it drops linearly to the Stern potential ψ_*d*_.^58^ The capacitance of the Stern layer is

$$C_{\mathrm{Stern}}=\frac{\sigma }{\psi_{0}-\psi_{d}}=\frac{\varepsilon \varepsilon_{0}}{d_{S}}$$ 3

where *d*_*S*_ is the Stern layer thickness. The slip plane with the ζ-potential is separated from the Stern plane by the sum of the effective hydrated radii of ions in the Stern layer and the bulk. In the diffuse layer, starting from the slip plane, ions can move freely along the surface.^59^ The potential drop in the diffuse layer follows the Poisson–Boltzmann equation.^60^ Solving it leads to the Grahame equation^61^ that connects the surface charge density with the potential at the Stern layer:

$$\sigma =\frac{2\varepsilon \varepsilon_{0}\kappa }{\beta e}\sinh\left(\frac{\beta e\psi_{d}}{2}\right)$$ 4

By combining Eqs. 2–4 and considering the relationship between the interfacial and bulk proton activities, given by the Boltzmann equation,^62^ as

$${\left[H^{+}\right]}_{\mathrm{int}}={\left[H^{+}\right]}_{\mathrm{bulk}}\exp\left(-\beta e\psi_{0}\right)$$ 5

we can derive a single equation for ψ_*d*_. It can be solved numerically when we introduce values for pH_bulk_, the Debye length, and the coverage densities of hydroxyl, amino, and dye groups (SI S5 Eq. S8).

To simplify this equation, we introduce the constants *C*_h‑d_ and *C*_a‑d_, which represent the ratios between hydroxyl and dye groups, and amino and dye groups. These constants can be used to determine the percentage of functionalization of the studied surfaces, and they serve as model parameters that will be used, along with other values, to determine the potentials of the EDL (SI S6). Importantly, the resulting percentages of functionalization provide a quantitative description of the surface chemistry, which allows us to ensure that the calibration method is applied consistently and reproducibly across substrates with different treatments. We determine these constants from the measured isoelectric points from the ζ-potential titrations, assuming that the surface charge density and surface potential are zero at these points. By doing so, we can rewrite Eq. S8 for ψ_*d*_ in terms of pH_bulk_, the Debye length, and the density of coverage of dye groups as follows:

$$\frac{2\varepsilon \varepsilon_{0}\kappa }{\beta e}\sinh\left(\frac{\beta e\psi_{d}}{2}\right)=\left(e\Gamma_{\mathrm{dye}}\right)\left(\frac{-C_{h‐d}}{1+D_{\mathrm{hydroxyl}}X_{1}}+\frac{C_{a‐d}}{1+D_{\mathrm{amino}}X_{2}}+\frac{-1}{1+D_{\mathrm{dye}}X_{1}}\right)$$ 6

where *X*_1_ and *X*_2_ are functions of *C*_Stern_, κ^–1^, and ψ_*d*_, and *D*_hydroxyl_, *D*_amino_, and *D*_dye_ are functions of *C*_Stern_ and the corresponding dissociation constants (SI S5 Eq. S16).

Eq. 6 cannot be solved analytically. We solve it numerically over a wide range of bulk pH values. To do this, we use *C*_Stern_ = 0.315 F/m^2^, in line with experimental measurements for a KCl electrolyte solution,^63,64^ and the calculated Debye length at each pH_bulk_ reported in Figure S2. The density of dye groups Γ_dye_ is set to 1/nm^2^ for all the studied samples, which is in the order of magnitude of the density of native hydroxyl groups and the substituting species, amino and dyes.^32,65^ We note that the calculation is not sensitive to the exact value of Γ_**dye**_.

After determining ψ_*d*_, we use this value to calculate the surface potential ψ_0_. Both values are shown in Figure 4a alongside the experimental measurement of the ζ-potential for each sample.

**Figure 4:**
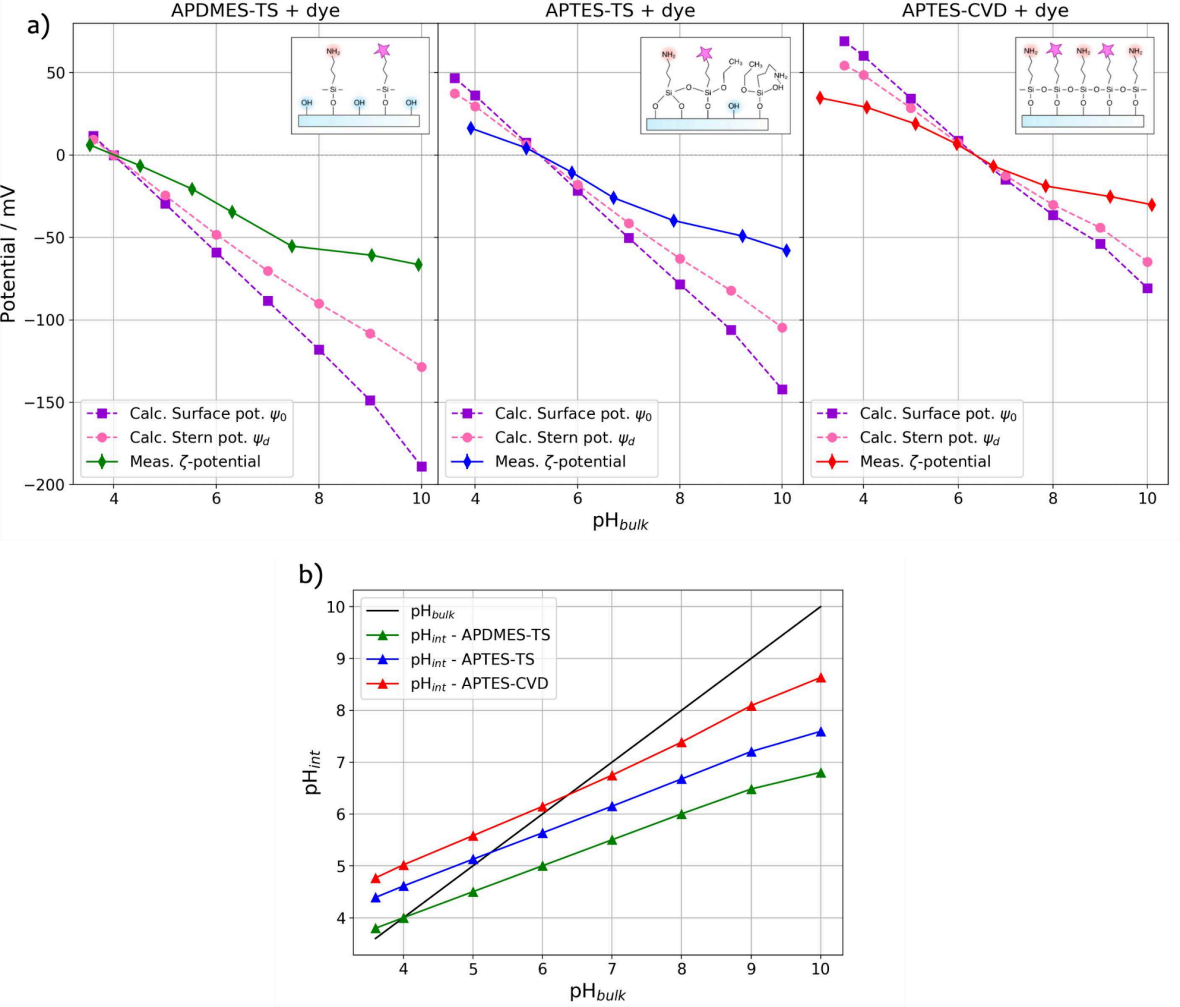
(a) Plots of the calculated potentials within the electric double layer showing the measured ζ-potential values for each sample (after dye grafting). (b) Plots of pH_int_ versus pH_bulk_ for APDMES-TS (green), APTES-TS (blue), and APTES-CVD (red) lines. Bulk pH is shown on a black line as a comparison reference.

The calculated potentials arising from the model agree well with our ζ-potential measurements. We note that, in general, the experimentally accessible ζ-potential is lower than the Stern potential solved by the model. The agreement confirms the model’s capability to predict the interfacial electrostatic environment on functionalized glass surfaces. The results underscore the interplay between surface substitution density and the electrostatic properties, as reflected by the surface and Stern potentials. Both potentials increase with higher substitution densities, attributable to enhanced surface charge densities derived from the varying functional group compositions.

A critical objective of this work is to understand and quantify the pH distribution across the electric double layer, particularly the interfacial pH. We use Eq. 5 to calculate interfacial pH values based on the surface potential ψ_0_. The corresponding pH_int_ values are shown in Figure 4b. The calculations reveal a narrowed pH range at the interface compared to the bulk, driven by the chemical equilibrium established by surface-bound species and the charge distribution within the double layer. The data indicates that surfaces with lower amino group density (e.g., APDMES-TS) exhibit more acidic interfacial pH values even in basic bulk environments. Conversely, increased substitution of hydroxyl groups reduces the discrepancy between bulk and interfacial pH, as the interfacial pH approaches the bulk value.

Our theoretical model and analysis show that, for glass surfaces functionalized with silanes and fluorescent dyes, the interfacial pH (pH_int_) follows an almost linear function of the bulk pH within the range of bulk pH values for which the ionic strength remained approximately constant, as defined by the experimental conditions of the ζ-potential titrations. For the specific electrolyte composition and ionic strength used in this work, this linear transformation was solved and validated within the pH range covered by our experiments. Building on this general finding, we introduce a simple method for quantitative in situ interfacial pH measurements. Importantly, the model is not limited to the specific pH range reported here, and we expect the linear relationship between interfacial and bulk pH to hold even beyond the range explored in this study.

To investigate the interfacial environment, we calibrate the fluorescent response of grafted dyes across varying bulk pH conditions (SI S7). We performed fluorescence measurements using an inverted confocal microscope. A buffer droplet was deposited on top of the functionalized surface, and fluorescence images of the interface were acquired around the contact line for different pH values, as shown in Figure 5a. The dye responds to local proton activity by varying its fluorescence intensity. The resulting fluorescence titration curves (Figure 5b), display a sigmoidal shape that resembles the trend described by the Henderson–Hasselbalch equation but differ slightly due to the influence of interfacial effects. These curves reflect the apparent response range of the dye on each surface. For reference, the dye used (pHrodo iFL Green STP Ester, ThermoFisher) has a dissociation constant of p*K* = 6.2 in free solution, which corresponds to a normalized fluorescence intensity of 0.5.

**Figure 5:**
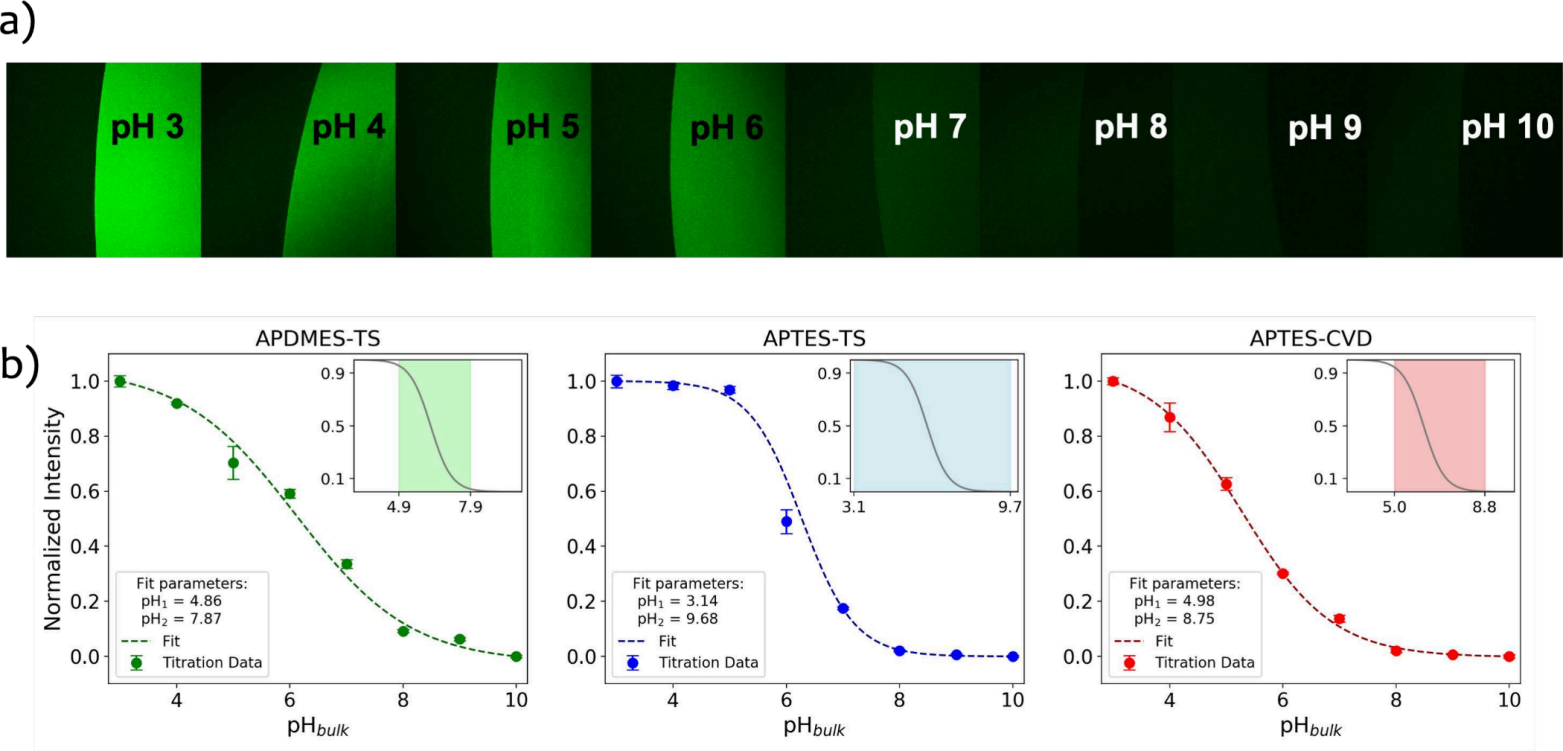
(a) Typical confocal microscopy images of the fluorescent titration experiment. Each square image is divided by the contact line of the droplet. The drop is placed on the right side of each square. (b) Fluorescence titration curves of the three functionalized surfaces, showing the normalized intensity of the dye as a function of pH_bulk_. The solid lines represent the best fit to a sigmoidal model used to extract the interfacial pH range (pH_1_, pH_2_), indicated by the shaded areas in the insets.

As predicted by the model, the interfacial pH (pH_int_) varies linearly within a narrowed range and shifts with the isoelectric point. Consistent with this behavior, the observed fluorescence titration curves also indicate a narrowed response range compared to the dye’s behavior in bulk solution, as shown in the subplots of Figure 5b. To quantitatively describe this behavior, we derive a transformation of the fluorescence intensity distribution expected under isolated conditions and fit this function to the titration data. From this analysis, the interfacial pH boundaries (pH_1_ and pH_2_) are extracted for each functionalization protocol, as summarized in Table 2. These boundaries reflect the interplay between functional group composition, electrostatic properties, and interfacial proton dynamics.

**Table 2:** Extracted pH Limits of the Interfacial pH

Aminosilane^a^	pH_1_	pH_2_
APDMES-TS	4.9	7.9
APTES-TS	3.1	9.7
APTES-CVD	5.0	8.8

a This corresponds to the aminosilane and silanization protocol used for each sample among the dye grafted on top.

The extracted interfacial pH limits provide a reliable basis for recalibrating the fluorescence signals through a simple linear transformation that relates the fluorescence titration range to the interfacial pH range. By normalizing the fluorescence intensity between its minimum and maximum values, each measured intensity can be associated with a corresponding value on the fitted titration curve. Specifically, the normalized fluorescence values — such as those shown in Figure 5b — are used to estimate the corresponding bulk pH via the inverse of the fitted function. The interfacial pH is then obtained by applying the linear transformation defined by the extracted interfacial pH boundaries (pH_1_ and pH_2_).

The linear relationship between pH_bulk_ and pH_int_ holds over a wide range of ionic strengths. Varying the ionic strength changes the range of the interfacial pH. This is reflected by the different pH_int_ ranges between our earlier theoretical predictions and the ones obtained in the calibration procedure. The ionic strengths of the electrolytes used for fluorescence titration were higher by a factor of ≈ 100 compared to the ζ-potential measurements and modeling. Additional deviations may arise from assumptions and approximations in the model, such as the uniformity of dye group density and idealized surface behavior. Future refinements to the model, incorporating experimental variability and improved surface characterization, are expected to enhance the accuracy of interfacial pH predictions.

To conclude, in this work we studied, both theoretically and experimentally, silica-based surfaces grafted with aminosilanes and functionalized with a pH-sensitive fluorescent dye. For these surfaces, the interfacial pH varies linearly with the bulk pH, though the interfacial range is narrowed compared to the bulk. We introduce a method for recalibrating the fluorescent response of the grafted dye that enables quantitative in situ measurements of the interfacial pH. This work bridges theoretical and experimental approaches to offer a robust tool for surface chemistry analysis. Although we employed pHrodo iFL Green STP Ester as a proof of concept, the method is not limited to this specific dye. It can be applied to any pH-sensitive fluorophore that exhibits a reversible, well-characterized protonation equilibrium and remains stable after surface immobilization (for example, fluorescein or rhodamine derivatives). In addition, the method is not restricted to a specific electrolyte. It should be implemented with buffer systems that maintain a well-defined and stable pH and are not significantly affected by external conditions. Because interfacial pH strongly influences a range of surface-driven phenomena, this method can support, for example, the development of pH-sensitive coatings in implantable or wearable biosensors and the characterization of switchable membrane surfaces whose charge or permeability is modulated by pH. Future applications may extend to environmental sensing, biomedical diagnostics, and materials science, where precise interfacial pH control is critical. Further refinement of the model and experimental protocols will enhance its predictive power and versatility in practical applications.

## Supplementary Material



## Notes

Sample preparation, AFM surface characterization, surface ζ-potential measurement, theoretical calculation of conductivity and Debye length, derivation of the model, model parameters, and fluorescence titration (PDF)
