# Supplementary material for: A Method for in Situ Interfacial pH Detection
Source: J Phys Chem Lett. 2025 Aug 21;16(34):8869–76. doi: 10.1021/acs.jpclett.5c02002 (PMC12400433; doi:10.1021/acs.jpclett.5c02002)
Supplement: Supplementary file 1 [file jz5c02002_si_001.pdf]

Supporting Information

A Method for in Situ Interfacial pH Detection

Karina N. Catalan, Aaron D. Ratschow, Hans-Jürgen Butt,* and Kaloian Koynov*

Max Planck Institute for Polymer Research, 55128 Mainz, Germany

E-mail: butt@mpip-mainz.mpg.de; koynov@mpip-mainz.mpg.de

S1 Sample preparation

Substrate cleaning: 25 mm circular cover glass slides No. 1.5 (0.16-0.19 mm) (D 263® M, Marienfeld) were sonicated for 5 min (each) in toluene (>= 99.8%, Fisher scientific), ethanol (absolute, VWR chemicals) and acetone (>= 99.5, Honeywell). Then the slides were dried using a nitrogen stream and subsequently activated and cleaned using 100% oxygen on a plasma cleaner (Femto BLS, Diener) for 10 min at 100% voltage (max. power) and 0.3 mbar of pressure.

Toluene solution (TS) silanization protocol: Cleaned and activated cover slips were immersed in a 1 wt% APDMES (95%, abcr) or 2 wt% APTES (99%, Sigma-Aldrich) solution prepared in anhydrous toluene (99.85%, Thermo Scientific). The solutions were contained in a Schlenk flask connected to an argon flow to prevent moisture ingress.[1] The mixture was stirred at 200 rpm for 20 min and 1 h, respectively. After the reaction, the functionalized cover slips were sonicated in toluene for 5 min, rinsed twice with ethanol, and dried under a nitrogen stream. The samples were then annealed at 110 °C for 30 min.

Chemical vapor deposition protocol (CVD): Cleaned and activated cover slips were placed on a ceramic plate inside a 150 mm desiccator (PP+PC, BRAND). On the bottom-center,

1 ml of APTES was placed in a container. The desiccator was evacuated to 50 mbar and left to react for 6 hours. Subsequently the desiccator was pumped continuously for 15 min to remove unreacted molecules.

Dye grafting: 1 mg of pHrodo™ Green STP Ester (Amino reactive, Invitrogen)[2] was dissolved in 100 ml of Dimethyl sulfoxide (DMSO), Spectrophotometric Grade (99.9+%, Thermo Scientific) to get a 10^{-5} M stock solution. The functionalized cover slips were placed on a Attofluor cell chamber (35 mm, Invitrogen) with an organic solvent-resistant O-ring and subsequently 400 μL of the dye stock solution was deposited on top of the cover slips and left to react for 1 hr in the dark. Later the samples were sonicated (5 min) and washed in DMSO (>=99.9%, Honeywell) and sonicated (5 min) and washed in ultrapure water. Lastly, the samples were dried under a nitrogen stream and then placed in a vacuum oven at 60 °C for 6 hours under vacuum.

S2 AFM surface characterization

Atomic force microscopy (AFM) measurements were performed with a JPK NanoWizard IV instrument operating in tapping mode, using ARROW-NCR probes (NanoAndMore) with a nominal tip radius below 10 nm. For each functionalized surface, three separate AFM images (Fig. S1) with a scan size of 5 × 5 μm² were acquired, and the root-mean-square roughness (S_q) values were averaged to obtain the reported values shown in Table S1.

Table S1: Root-mean-square roughness (Sq) of the three functionalized glass surfaces, measured by AFM (5 × 5 μm² scans, three images per sample, averaged). Values are reported in picometers (pm) with standard deviations.

	S_q - pm
APDMES-TS + dye	294.0 ± 6.5
APTES-TS + dye	245.0 ± 14.5
APTES-CVD + dye	300.0 ± 8.2

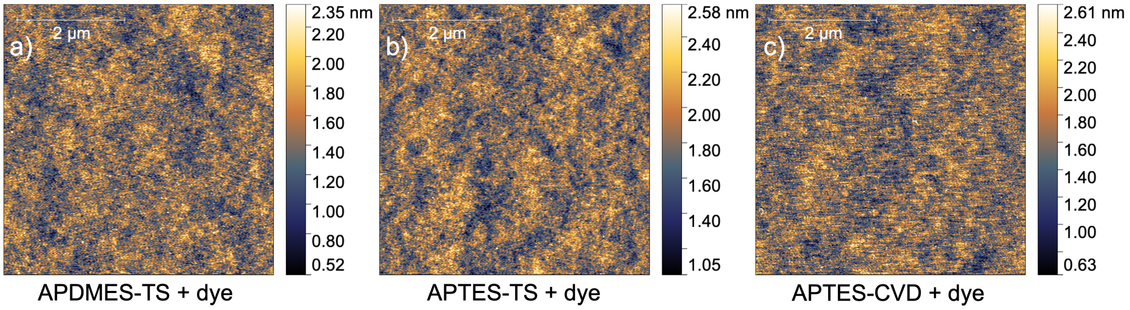

Figure S1: AFM topography images (5×5 µm^2 scan size) of the three functionalized glass surfaces: (a) APDMES-TS + dye, (b) APTES-TS + dye, and (c) APTES-CVD + dye. The color scales indicate height in nanometers.

S3 Surface ζ-potential measurement

Zeta potential titration measurements are conducted using an electrokinetic analyzer for solid surfaces (SurPASS 3, Anton Paar).[3] The pH scan function determines zeta potential values across a range from approximately pH 3 to 10, in increments of 1. The conductivity is initially set to 12.5 mS/m at pH 10 by titrating with 50 mM KCl. Subsequently, lower pH values are achieved by titrating with 50 mM HCl, while maintaining conductivity within the range of 10 mS/m, as shown in transparent dotted lines in Fig. S2. Each measurement is performed in triplicate and averaged for final presentation.

S4 Theoretical calculation of Conductivity and Debye length

We begin by defining the initial state of the electrolyte, which is titrated with KOH until reaching pH 10. Subsequently, KCl is added to adjust the conductivity to 12 mS/m. The conductivity of an electrolyte can be calculated from the concentrations of the various ions present in the solution and their respective molar conductivities at infinite dilution, as follows:

$$\kappa = \lambda^0_{\mathrm{K}^+}[\mathrm{K}^+] + \lambda^0_{\mathrm{OH}^-}[\mathrm{OH}^-] + \lambda^0_{\mathrm{H}^+}[\mathrm{H}^+] + \lambda^0_{\mathrm{Cl}^-}[\mathrm{Cl}^-] \tag{S1}$$

This expression allows us to determine the initial concentration of KCl (i.e., the salt

contribution) present in the solution, under the assumption that $[K^+]_{salt} = [Cl^-]_{salt}$. We further assume that $[K^+]_{KOH} = [OH^-] = 10^{14-pH}$ M and that initially $[H^+] = 0$. Substituting these values into Eq. (S1) yields:

$$12.0 = \lambda^0_{K^+}\left([K^+]_{salt} + 10^{-4}\text{ M}\right) + \lambda^0_{OH^-}\cdot 10^{-4}\text{ M} + \lambda^0_{H^+}\cdot 0 + \lambda^0_{Cl^-}[Cl^-]_{salt} \tag{S2}$$

Using the molar conductivities at infinite dilution:[4] $\lambda^0_{K^+} = 73.5 \times 10^{-4}$ S·m²/mol, $\lambda^0_{OH^-} = 198.6 \times 10^{-4}$ S·m²/mol, $\lambda^0_{H^+} = 349.8 \times 10^{-4}$ S·m²/mol, and $\lambda^0_{Cl^-} = 76.3 \times 10^{-4}$ S·m²/mol, we solve Eq. (S2) to determine the initial salt concentration:

$$[K^+]_{salt} = [Cl^-]_{salt} = 0.619\text{ mM}$$

With this value, we calculate the ionic strength (I) of the electrolyte using its definition:

$$I = \frac{1}{2}\left([K^+]z^2_K + [Cl^-]z^2_{Cl} + [OH^-]z^2_{OH} + [H^+]z^2_H\right) \tag{S3}$$

where $z_{K^+} = +1$, $z_{OH^-} = -1$, $z_{H^+} = +1$, and $z_{Cl^-} = -1$. By substituting the respective concentrations into Eq. (S3), we calculate the ionic strength at pH 10 and a conductivity of 12 mS/m as:

$$I = 0.719\text{ mM}$$

With the ionic strength known, we calculate the Debye length for a monovalent electrolyte using the expression:

$$\kappa^{-1} = \sqrt{\frac{\varepsilon_r\varepsilon_0 k_B T}{2N_A e^2 I}} \tag{S4}$$

This yields a Debye length of:

$$\kappa^{-1} = 11.3\text{ nm}$$

Next, we calculate the values for the conductivity, ionic strength, and Debye length for the following pH values (decreasing) down to 3.6 in steps of 1. From pH 9 onward, the order of

the calculations differs from that at pH 10, since the unknown quantity is the concentration of HCl used to titrate the solution to pH 9. This concentration can be determined by considering that the added HCl introduces a number of protons (H^+) sufficient to neutralize the existing OH^- ions (by forming H_2O), resulting in the target OH^- concentration corresponding to pH 9, which is 10^{-5} M.

$$10^{-4} - [\mathrm{Cl}^-]_{\mathrm{HCl}} = 10^{-5} \rightarrow [\mathrm{Cl}^-]_{\mathrm{HCl}} = 9 * 10^{-5}\ \mathrm{M}$$

With this, all ion concentrations at pH 9 are known, allowing the calculation of the desired parameters:

$$\begin{aligned}
[\mathrm{K}^+] &= [\mathrm{K}^+]_{\mathrm{salt}} + [\mathrm{K}^+]_{\mathrm{KOH}} = 0.619\ \mathrm{mM} + 10^{-4}\ \mathrm{M} \\
[\mathrm{OH}^-] &= 10^{-5} \\
[\mathrm{H}^+] &= 0 \\
[\mathrm{Cl}^-] &= 0.619\ \mathrm{mM} + 9 * 10^{-5}\ \mathrm{M}
\end{aligned}$$

$$\kappa = 10.9\ \mathrm{mS/m}, \quad I = 0.719\ \mathrm{mM}, \quad \kappa^{-1} = 11.3\ \mathrm{nm}$$

By continuing this procedure, we calculate the parameters for all pH values. These values are listed in Table S2, and the calculated conductivity and Debye length are shown in Fig. S2.

Table S2: Calculated values of conductivity (κ), ionic strength (I) and Debye length (κ^{-1}) for the electrolyte used in the surface ζ-potential titrations, as a function of the bulk pH.

pH	κ - mS/m	I - mM	κ^{-1} - nm
10	12.0	0.719	11.3
9	10.9	0.719	11.3
8	10.8	0.719	11.3
7	10.8	0.719	11.3
6	10.8	0.720	11.3
5	11.2	0.729	11.3
4	15.0	0.819	10.6
3.6	21.5	0.971	9.75

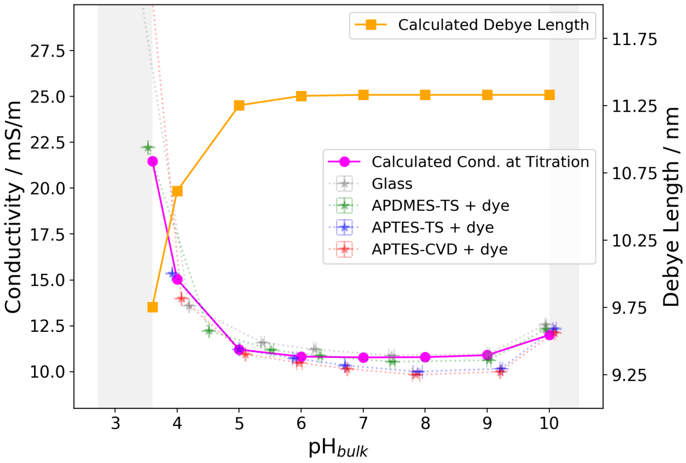

Figure S2: Measured conductivity during titration experiments starting from pH 10 (transparent lines) and calculated conductivity for a KCl solution titrated with 50 mM HCl, starting at 12 mS/m. The corresponding Debye length κ^{-1} is also shown.

S5 Derivation of the model

Let us consider a glass surface functionalized with aminosilanes and a pH-sensitive dye. Three different chemical species are present, and they dissociate upon contact with an electrolyte as follows:

$$\begin{aligned} \mathrm{S-OH} &\rightleftharpoons \mathrm{S-O^- + H^+ (hydroxyl)} \\ \mathrm{S-NH_3^+} &\rightleftharpoons \mathrm{S-NH_2 + H^+ (amino)} \\ \mathrm{S-DH} &\rightleftharpoons \mathrm{S-D^- + H^+ (dye)} \end{aligned}$$

, where $\mathrm{D^-}$ and DH are the depotronated and protonated forms of the dye respectively.

Since the roughness of the functionalized surfaces is extremely low (below 350 pm, see AFM data in Section S2), we treat the chemical species as strictly localized on the surface of the glass and apply the Stern model. Based on the measured Stern capacitance ($C_{\mathrm{Stern}} =$ 0.315 F/m), the effective thickness of the Stern layer is approximately 2.2 nm. Because this thickness is far greater than the measured roughness, any positional fluctuations of the dye molecules due to surface topography or conformational changes are negligible at this scale.

The surface charge density (σ) is therefore given by the contribution of all charged groups:

$$\sigma = -e\Gamma_{\mathrm{O^-}} + e\Gamma_{\mathrm{NH_3^+}} - e\Gamma_{\mathrm{D^-}} \tag{S5}$$

Here, Γ accounts for the concentration of the specific group. In general, it is expected that $\Gamma_{\mathrm{hydroxyl}}$, Γ_{amino}, and Γ_{dye} which are the sum of protonated and deprotonated forms of a functional group is kept constant in a given system.

$$\Gamma_{\mathrm{Total}} = (\Gamma_{\mathrm{O^-}} + \Gamma_{\mathrm{OH}}) + (\Gamma_{\mathrm{NH_3^+}} + \Gamma_{\mathrm{NH_2}}) + (\Gamma_{\mathrm{D^-}} + \Gamma_{\mathrm{DH}}) = \Gamma_{\mathrm{hydroxyl}} + \Gamma_{\mathrm{amino}} + \Gamma_{\mathrm{dye}}$$

The density of charged groups of a specific functional group will vary according to its acid dissociation constant (pK) and proton activity on its vicinity ($\left[\mathrm{H^+}\right]_{\mathrm{int}}$) and it is given by the mass reaction law for deprotonation reactions:

$$\underbrace{\mathrm{HA}}_{\mathrm{acid}} \rightleftharpoons \underbrace{\mathrm{A^-}}_{\mathrm{base}} + \mathrm{H^+}$$

$$\frac{[\mathrm{H^+}]_{\mathrm{int}}\Gamma_{\mathrm{A^-}}}{\Gamma_{\mathrm{HA}}} = 10^{-\mathrm{pK}}\mathrm{M} \tag{S6}$$

The proton activity (related to the pH via $[\mathrm{H^+}] = 10^{-\mathrm{pH}}\mathrm{M}$) at the interface is connected to the bulk pH and to the surface electrochemical potential by the Boltzmann equation:

$$[\mathrm{H^+}]_{\mathrm{int}} = [\mathrm{H^+}]_{\mathrm{bulk}} \exp\left(-\beta e \psi_0\right) \tag{S7}$$

, where $\beta = (k_B T)^{-1}$ accounts for the thermal energy.

We can now express the surface charge density in terms of the density of each ionizable

group, its pK, the bulk pH and the surface potential by combining Eqs. (S5)–(S7):

$$\begin{aligned}
\Gamma_{\text{hydroxyl}} &= \Gamma_{\text{O}^-} + \Gamma_{\text{OH}} = \Gamma_{\text{O}^-}(1 + 10^{-(\text{pH}_{\text{bulk}} - \text{pK}_{\text{hydroxyl}})} \exp(-\beta e \psi_0)) \\
\Gamma_{\text{amino}} &= \Gamma_{\text{NH}_2} + \Gamma_{\text{NH}_3^+} = \Gamma_{\text{NH}_3^+}(1 + 10^{(\text{pH}_{\text{bulk}} - \text{pK}_{\text{amino}})} \exp(\beta e \psi_0)) \\
\Gamma_{\text{dye}} &= \Gamma_{\text{D}^-} + \Gamma_{\text{DH}} = \Gamma_{\text{D}^-}(1 + 10^{-(\text{pH}_{\text{bulk}} - \text{pK}_{\text{dye}})} \exp(-\beta e \psi_0))
\end{aligned}$$

$$\sigma = \frac{-e\Gamma_{\text{hydroxyl}}}{1 + 10^{-(\text{pH}_{\text{bulk}} - \text{pK}_{\text{hydroxyl}})} e^{-\beta e \psi_0}} + \frac{e\Gamma_{\text{amino}}}{1 + 10^{(\text{pH}_{\text{bulk}} - \text{pK}_{\text{amino}})} e^{\beta e \psi_0}} + \frac{-e\Gamma_{\text{dye}}}{1 + 10^{-(\text{pH}_{\text{bulk}} - \text{pK}_{\text{dye}})} e^{-\beta e \psi_0}} \tag{S8}$$

In order to reduce the number of variables in Eq. (S8), we proceed to use the isoelectric points (pH at which the ζ-potential is zero) extracted from the surface ζ-potential measurements and substitute them to conveniently find the ratio of substitution between the chemical species present at the surface. Two different isoelectric points are measured per silanization protocol, one before dye grafting (pI_1) and one after dye grafting (pI_2). For simplification, we assume the samples to be homogeneous and the ionic strength of the electrolyte to be low enough that allow us to set the net charge density and the surface potential to zero at the isoelectric points. The first case we will analyze will be a substitution of 1:1 between hydroxyl:amino groups. Let us substitute the pertinent data in Eq. (S8):

$$0 = \frac{-e\Gamma_{\text{hydroxyl}}}{1 + 10^{-(\text{pI}_1 - \text{pK}_{\text{hydroxyl}})}} + \frac{e\Gamma_{\text{amino}^*}}{1 + 10^{(\text{pI}_1 - \text{pK}_{\text{amino}})}}$$

For simplicity, we introduce the following constants:

$$A = 1 + 10^{-(\text{pI}_1 - \text{pK}_{\text{hydroxyl}})} \qquad B = 1 + 10^{(\text{pI}_1 - \text{pK}_{\text{amino}})} \qquad C = 1 + 10^{-(\text{pI}_2 - \text{pK}_{\text{hydroxyl}})}$$

$$D = 1 + 10^{(\text{pI}_2 - \text{pK}_{\text{amino}})} \qquad E = 1 + 10^{-(\text{pI}_2 - \text{pK}_{\text{dye}})}$$

Now we can express the density of amino groups as a fraction of the density of hydroxyl groups.

$$\frac{\Gamma_{\text{amino}^*}}{\Gamma_{\text{hydroxyl}}} = \frac{1 + 10^{-(\text{pI}_1 - \text{pK}_{\text{amino}})}}{1 + 10^{(\text{pI}_1 - \text{pK}_{\text{hydroxyl}})}} = \frac{B}{A} \tag{S9}$$

The surface density Γ_{amino^*} is the density of amino groups before grafting the dye and following the substitution rule should be equal to the amino groups that didn't react with the dye plus the dye groups.

$$\Gamma_{\text{Total}} = \Gamma_{\text{hydroxyl}} + \Gamma_{\text{amino}^*} = \Gamma_{\text{hydroxyl}} + \Gamma_{\text{amino}} + \Gamma_{\text{dye}} \tag{S10}$$

Let us examine now the expression obtained for the sample after the dye grafting:

$$\begin{aligned} 0 &= \frac{-e\Gamma_{\text{hydroxyl}}}{1+10^{-(\text{pI}_2-\text{pK}_{\text{hydroxyl}})}} + \frac{e\Gamma_{\text{amino}}}{1+10^{(\text{pI}_2-\text{pK}_{\text{amino}})}} + \frac{-e\Gamma_{\text{dye}}}{1+10^{-(\text{pI}_2-\text{pK}_{\text{dye}})}} \\ 0 &= \frac{-\Gamma_{\text{hydroxyl}}}{C} + \frac{\Gamma_{\text{amino}^*}-\Gamma_{\text{dye}}}{D} + \frac{-\Gamma_{\text{dye}}}{E} \end{aligned}$$

By substituting equation Eq. (S9) we get a relation between hydroxyl and dye groups.

$$\frac{\Gamma_{\text{hydroxyl}}}{C} - \frac{\Gamma_{\text{hydroxyl}}}{D}\cdot\frac{B}{A} = \frac{-\Gamma_{\text{dye}}}{D} + \frac{-\Gamma_{\text{dye}}}{E}$$

For simplicity we will define this constant ratio as $C_{\text{h-d}}$.

$$\frac{\Gamma_{\text{hydroxyl}}}{\Gamma_{\text{dye}}} = \frac{C\cdot A(E+D)}{E(C\cdot B-D\cdot A)} \equiv C_{\text{h-d}} \tag{S11}$$

By replacing equation Eq. (S11) and Eq. (S10) on Eq. (S9) we can get an expression for the ratio between amino and dye groups:

$$\frac{\Gamma_{\text{amino}^*}}{\Gamma_{\text{hydroxyl}}} = \frac{\Gamma_{\text{amino}}+\Gamma_{\text{dye}}}{\Gamma_{\text{hydroxyl}}} = \frac{\Gamma_{\text{amino}}+\Gamma_{\text{dye}}}{\Gamma_{\text{dye}}\cdot C_{\text{h-d}}} = \frac{B}{A}$$

$$\frac{\Gamma_{\text{amino}}}{\Gamma_{\text{dye}}} = \frac{B\cdot C_{\text{h-d}}}{A} - 1 = \frac{C\cdot B(E+D)}{E(C\cdot B-D\cdot A)} - 1 = \frac{C\cdot B\cdot D+E\cdot D\cdot A}{E(C\cdot B-D\cdot A)}$$

$$\frac{\Gamma_{\text{amino}}}{\Gamma_{\text{dye}}} = \frac{D(C\cdot B+E\cdot A)}{E(C\cdot B-D\cdot A)} \equiv C_{\text{a-d}} \tag{S12}$$

By substituting equation Eq. (S11) and Eq. (S12) into Eq. (S8) we can rewrite the surface charge density in terms of the dye surface density.

$$\begin{aligned}\sigma &= \frac{-e\Gamma_{\text{dye}}C_{\text{h-d}}}{1+10^{-(\text{pH}_{\text{bulk}}-\text{pK}_{\text{hydroxyl}})}e^{-\beta e\psi_0}} + \frac{e\Gamma_{\text{dye}}C_{\text{a-d}}}{1+10^{(\text{pH}_{\text{bulk}}-\text{pK}_{\text{amino}})}e^{\beta e\psi_0}} + \frac{-e\Gamma_{\text{dye}}}{1+10^{-(\text{pH}_{\text{bulk}}-\text{pK}_{\text{dye}})}e^{-\beta e\psi_0}} \\ &= (e\Gamma_{\text{dye}})\left(\frac{-C_{\text{h-d}}}{1+10^{-(\text{pH}_{\text{bulk}}-\text{pK}_{\text{hydroxyl}})}e^{-\beta e\psi_0}} + \frac{C_{\text{a-d}}}{1+10^{(\text{pH}_{\text{bulk}}-\text{pK}_{\text{amino}})}e^{\beta e\psi_0}} + \frac{-1}{1+10^{-(\text{pH}_{\text{bulk}}-\text{pK}_{\text{dye}})}e^{-\beta e\psi_0}}\right)\end{aligned} \tag{S13}$$

This last expression still contains two unknown variables: ψ_0 and σ. To reduce it to a single variable, we consider the equations governing the electrostatic equilibrium at the electrical double layer (EDL), particularly those described by the Gouy–Chapman–Stern model.

The Stern model considers that counterions are separated from the surface by a distance d_S, which is in general given by the ionic radius of the electrolyte. In this region, known as the Stern layer the potential drops linearly from the surface electrostatic potential ψ_0 to the potential at the Stern plane, ψ_d.

$$C_{\text{Stern}} = \frac{\sigma}{\psi_0 - \psi_d} = \frac{\varepsilon\varepsilon_0}{d_S} \tag{S14}$$

Further away from the surface lies the diffuse layer, where the distribution of mobile ions is governed by the Poisson–Boltzmann (PB) equation. A useful result derived from solving it in this region is the Grahame equation, which relates the surface charge density of an isolated, flat interface to the electrostatic potential and the ionic strength of the solution.

$$\sigma = \frac{2\varepsilon\varepsilon_0\kappa}{\beta e}\sinh(\frac{\beta e\psi_d}{2}) \tag{S15}$$

To reduce Eq. (S13) to a single variable, the potential at the Stern plane ψ_d, we combine the expressions for the Stern layer capacitance and the surface charge density, given by equations Eq. (S14) and Eq. (S15), respectively. This substitution links the electrostatic potential drop across the Stern layer with the ionic distribution in the diffuse layer, providing

a self-consistent expression for ψ_d.

$$\sigma = \frac{2\varepsilon\varepsilon_0\kappa}{\beta e}\sinh(\frac{\beta e\psi_d}{2}) \qquad C_{\text{Stern}} = \frac{\sigma}{\psi_0 - \psi_d} \qquad \Rightarrow \qquad \psi_0 = \frac{\sigma}{C_{\text{Stern}}} + \psi_d = \frac{2\varepsilon\varepsilon_0\kappa}{C_{\text{Stern}}\beta e}\sinh(\frac{\beta e\psi_d}{2}) + \psi_d$$

By substituting the expression for σ and ψ_0 we obtain:

$$\frac{2\varepsilon\varepsilon_0\kappa}{\beta e}\sinh(\frac{\beta e\psi_d}{2}) = (e\Gamma_{\text{dye}})\left(\frac{-C_{\text{h-d}}}{1 + D_{\text{hydroxyl}}X_1} + \frac{C_{\text{a-d}}}{1 + D_{\text{amino}}X_2} + \frac{-1}{1 + D_{\text{dye}}X_1}\right) \tag{S16}$$

where:

$$X_1 = \exp\left(-\frac{1}{C_{\text{Stern}}}\left(2\varepsilon\varepsilon_0\kappa\sinh(\tfrac{\beta e\psi_d}{2}) + e\beta C_{\text{Stern}}\psi_d\right)\right)$$
$$X_2 = \exp\left(\frac{1}{C_{\text{Stern}}}\left(2\varepsilon\varepsilon_0\kappa\sinh(\tfrac{\beta e\psi_d}{2}) + e\beta C_{\text{Stern}}\psi_d\right)\right)$$
$$D_{\text{dye}} = 10^{-(\text{pH}_{\text{bulk}} - \text{pK}_{\text{dye}})} \qquad D_{\text{hydroxyl}} = 10^{-(\text{pH}_{\text{bulk}} - \text{pK}_{\text{hydroxyl}})} \qquad D_{\text{amino}} = 10^{(\text{pH}_{\text{bulk}} - \text{pK}_{\text{amino}})}$$

After numerically solving for ψ_d using the parameters Γ_{dye}, C_{Stern}, pK_i, and κ as a function of pH_{bulk}, we can then work backwards to calculate ψ_0, σ, and the interfacial pH. Altogether, this model captures both the chemical and electrostatic equilibria at the interface, allowing us to quantitatively link surface composition, ionization states, and potential profiles to external conditions such as bulk pH and ionic strength.

S6 Model parameters

The model requires several parameters as input, which must be either predefined or calculated prior to numerically solving the Stern potential (ψ_d) for a given bulk pH. The parameters that are independent of the analyzed sample are listed separately in Table S3, while those that vary with the specific sample are provided in Table S4.

From the $C_{\text{h–d}}$ and $C_{\text{a–d}}$ values extracted from the titration measurements we can further determine the percentage of functionalization $\theta(\%)$ by means of the definition of the ratios

expressed in Eq. (S11) and Eq. (S12) as it follows,

$$\frac{\theta_{\text{hydroxyl}}}{100} = \frac{\Gamma_{\text{hydroxyl}}}{\Gamma_{\text{Total}}} = \frac{C_{\text{h-d}}}{C_{\text{h-d}} + C_{\text{a-d}} + 1} \qquad \frac{\theta_{\text{amino}}}{100} = \frac{\Gamma_{\text{amino}}}{\Gamma_{\text{Total}}} = \frac{C_{\text{a-d}}}{C_{\text{a-d}} + C_{\text{h-d}} + 1}$$

$$\frac{\theta_{\text{dye}}}{100} = \frac{\Gamma_{\text{dye}}}{\Gamma_{\text{Total}}} = \frac{1}{1 + C_{\text{h-d}} + C_{\text{a-d}}}$$

Table S3: Parameters that remain constant across all studied samples. These values include parameters obtained from the literature, estimated values, and parameters calculated in this work.

$\varepsilon\varepsilon_0$	β	$\text{pK}_{\text{hydroxyl}}$	pK_{amino}	pK_{dye}	C_{Stern}	Γ_{dye}
F/m	J^{-1}	-	-	-	F/m^2	nm^{-2}
$78.4 \times \varepsilon_0$	$(k_B \cdot (298\text{ K}))^{-1}$	7.5	9.7	6.2	0.315	1

Table S4: Parameters as a function of bulk pH — which determines the Debye length (κ^{-1}) — that vary depending on the specific sample. These values include the ratios of chemical species ($C_{\text{h–d}}$ and $C_{\text{a–d}}$) calculated from their definitions given in Eq. (S11) and Eq. (S12).

		APDMES-TS + dye		APTES-TS + dye		APTES-CVD + dye	
pH	κ^{-1} - nm	$C_{\text{h-d}}$	$C_{\text{a-d}}$	$C_{\text{h-d}}$	$C_{\text{a-d}}$	$C_{\text{h-d}}$	$C_{\text{a-d}}$
3.6	9.75	1752	0.5601	115.7	0.7651	8.970	1.198
4	10.6						
5	11.3						
6	11.3						
7	11.3						
8	11.3						
9	11.3						
10	11.3						

Using the values listed on Table S4 we proceed to calculate the percentage of functionalization of the studied samples shown in Table S5. These percentages provide a quantitative assessment of the functionalization state of each surface, ensuring that the calibration method can be compared across samples with different treatments. This directly addresses reproducibility and consistency, as surfaces with comparable functionalization ratios yield consistent calibration behavior.

Table S5: Percentages of functionalization of the different chemical species present in the different analyzed samples.

APDMES-TS + dye			APTES-TS + dye			APTES-CVD + dye		
θ_{hydroxyl}	θ_{amino}	θ_{dye}	θ_{hydroxyl}	θ_{amino}	θ_{dye}	θ_{hydroxyl}	θ_{amino}	θ_{dye}
99.91	0.03	0.06	98.50	0.65	0.85	80.32	10.73	8.95

S7 Fluorescence titration

To assess the response of the immobilized dye, fluorescence titration was carried out by depositing 3 μL drops of buffer solutions (pH 3 to 10, ROTI Calipur buffer solutions, ROTH) onto the functionalized surfaces. Three images of 225 μm $\times$ 225 μm were acquired per titration point using a confocal laser scanning microscope (CLSM, LSM 510, Zeiss) equipped with a C-Apochromat 40x/1.2 W water immersion objective (Zeiss). The excitation was done with the 488 nm line of an Argon laser fiber coupled to the microscope. The fluorescence emission was detected in the range 535 to 590 nm. In parallel to the fluorescence, reflection signal was also detected, as shown in Fig. S3a. The excitation laser intensity was kept constant at about 100 μW after the objective. Particular care was taken to focus the confocal plane precisely on the dye layer for each acquisition. Fluorescence and reflection channels were recorded separately for subsequent analysis Fig. S3b.

The reflection channel was used to identify the contact region between the buffer drop and the surface. Fluorescence intensity was then extracted from this region using ImageJ. For each image, the mean intensity was measured within three randomly selected squares (each 120×120 pixels) located in the drop–surface contact area. These three measurements were averaged to obtain a single intensity value per image. For each titration point, three such images were analyzed, and their averaged intensity values were further averaged to yield a final mean intensity per titration point. These values were normalized using min-max normalization based on the minimum and maximum intensity values across the pH range to account for signal variability. The normalized values were then plotted as a function of bulk pH. The resulting data were fitted using a transformed form of the Henderson–Hasselbalch

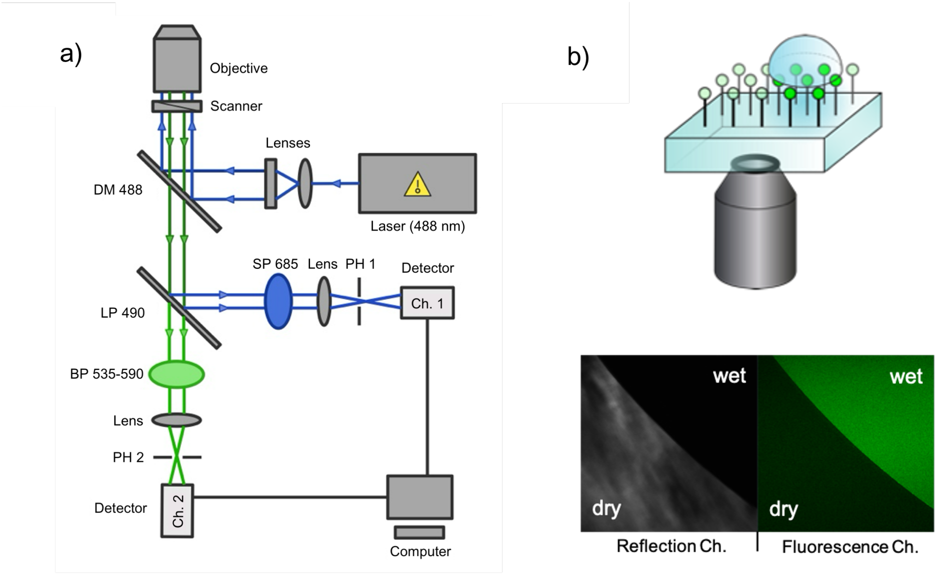

Figure S3: a) Schematic of the beam path used in the CLSM to split images into two channels. b) Reflection and fluorescence images of a pH 5 buffer drop deposited on a labeled surface.

equation, as shown in Eq. (S17), to extract the interfacial pH range, which exhibits a linear relationship with the bulk pH.

$$\mathrm{I_{nor}}(\mathrm{pH_{bulk}}) = a \cdot \left(\frac{1}{1+10^{c-\mathrm{p}K_a}}\right) + b \tag{S17}$$

, where:

$$a = \frac{(1+10^{\mathrm{pH_1-pK}})(1+10^{\mathrm{pH_2-pK}})}{(1+10^{\mathrm{pH_2-pK}})-(1+10^{\mathrm{pH_1-pK}})}$$
$$b = -\frac{(1+10^{\mathrm{pH_1-pK}})}{(1+10^{\mathrm{pH_2-pK}})-(1+10^{\mathrm{pH_1-pK}})}$$
$$c = \frac{(\mathrm{pH_{bulk}}-3)(\mathrm{pH_2}-\mathrm{pH_1})}{7} + \mathrm{pH_1}$$

References

(1) Millar, S. Tips and Tricks for the Lab: Air-Sensitive Techniques (3). ChemViews Magazine, 2013; https://doi.org/10.1002/chemv.201300042, Published July 2, 2013.

(2) Thermo Fisher Scientific pHrodo™ iFL STP Ester Dyes User Guide. User Manual, Pub. No. MAN0017101, Rev. A.00, 2021; https://assets.fishersci.com/TFS-Assets/LSG/manuals/MAN0017101_pHrodo_iFL_STP_dyes_UG.pdf, Accessed May 9, 2025.

(3) Luxbacher, T.; Bukvsek, H.; Petrinic, I.; Puvsi'c, T. Zeta potential determination of flat solid surfaces using a SurPASS electrokinetic analyzer. *Tekstil: Journal of Textile Clothing Technology* **2009**, *58*.

(4) Bard, A. J.; Faulkner, L. R. *Electrochemical Methods: Fundamentals and Applications*, 2nd ed.; John Wiley & Sons: New York, 2001.
